# News and Miscellany

**Published:** 1901-10

**Authors:** 


### News and Miscellany.

**Dr. L. G. C. Buchanan** has removed from Vaughn to San Angelo, Tex.

**Dr. Theodore Buehring** has removed from Ottine, Tex., to Nordheim, Tex.

**The distinguished Prof. Rudolf Virchow** will celebrate his 80th birthday on October 13.

**For Sale.**—An unopposed cash practice for price of property. Physician wishes to retire. Address "Physician" (with stamp), care of this office.

**Married,** at Galveston, September 25, ult., Dr. Thomas Lubbock Kennedy to Miss Nora, daughter of Mr. and Mrs. Theodore Kent Thompson, all of Galveston.

**Dr. Jno. Punton,** of the University Medical College, Kansas City, and editor of the sprightly *Kansas City Medical Index-Lancet*, has returned from a two months trip in Europe.

**Prof. Bacon Saunders, M. D.,** Dean of the Medical Department Fort Worth University, was elected Vice-President of the National Association of Railway Surgeons at their June meeting at St. Paul.

**Dr. E. V. Hamilton,** of Austin, surgeon in charge of city and county hospital has just returned from Chicago, where he took a post graduate course at the Post Graduate Medical School, 2400 Dearborn street.

**The Editor of this paper** strongly endorses the action of General Charles H. Grosvenor, in requiring a certain share of the proceeds from the sale of his book to be set aside for a McKinley Monument Fund. Our readers will see an advertisement of this book in another column of this paper.

**Splendid location** for a physician in rich and prosperous section of South Texas, can be secured by purchasing residence, office, stables, etc., and a cottage on separate lot. Practice realizes $3,500 to $6,000 yearly. Price of property, $3,200; $1,000 cash, balance on accommodating terms. Address F. W., care TEXAS MEDICAL JOURNAL, Austin, Texas.

---

**Under the Auspices of the Chicago Medical Society** a banquet and celebration in honor of Dr. Nathan Smith Davis, M. D., LL. D., who is the oldest living president of the society and widely known and honored among the profession by his long connection with the American Medical and other associations, was given at the Auditorium Hotel, Chicago, Saturday evening, October 5, 1901.

---

**Dr. Franklin Jerome Hall,** of Dallas, Texas, was married on August 28th to Miss Rowena Mary, daughter of Dr. and Mrs. Frederick William Russel, of Winchendon, Mass. Dr. Hall is prominent in North Texas as an eye and ear specialist and his bride is the daughter of a prominent specialist on nervous diseases in his native State. Dr. and Mrs. Hall will reside in Dallas.

**University of Dallas**—Medical Department: Dr. S. H. Stout, President of the Advisory Board of this University, calls our attention to the establishment of the Departments of Medicine and Pharmacy, and announces the purpose of the University to establish those of Dentistry and Law. Dr. Stout was the Medical Director of Hospitals of the Southern Confederacy. Write to him for a copy of circular letter of September 14, ult.

---

**New Orleans Polyclinic.**—Physicians will find the Polyclinic an excellent means for posting themselves upon modern progress in all branches of medicine and surgery. The specialties are fully taught, particularly laboratory work. Fifteenth annual session opens November 14, 1901. For further information address Dr. Isadore Dyer, Secretary, New Orleans Polyclinic, New Orleans.

---

**Examinations for Army Medical Department.**—The examination for applicants for appointments as Assistant Surgeon in the army has been resumed in Washington and San Francisco; the Army Medical Boards convened in those cities will remain in session so long as there are candidates to be examined. Seventy-six vacancies in the Medical Department still remain to be filled, and as it is desired by the military authorities that the department be filled up to its full legal limit as early as practicable, all eligible applicants will be afforded opportunity for examination; those qualified will be commissioned at an early date. Full information as to eligibility, nature and scope of examination, etc., may be obtained upon application to the Surgeon General, U. S. Army, Washington, D. C.

---

**Died,** in Victoria, Texas, September 14, ult., Dr. W. G. Thornton of that city.

At a meeting of the physicians of Victoria a committee was appointed to draft suitable resolutions to express the esteem in which Dr. Thornton was held by the profession. The following was reported and approved:

*Resolved*, That we recognized in Dr. Thornton a man of intelligence and integrity and a physician of high professional honor and skill. We testify to his courteousness and fair dealing in our profession and to his charity and efficiency in his practice. It was pleasant to be associated with him, either in the sick room or in

### BANKRUPT VITALITY

—be it manifested as nervous exhaustion, malnutrition, or anæmia—is the condition that

### GRAY'S Glycerine TONIC Comp.

speedily changes to vigor of nerves, blood and tissues.

THE PURDUE FREDERICK CO.,

No. 15 Murray Street, New York.

social intercourse, and we will remember him with feelings of admiration and esteem. We tender to the bereaved family our sincere sympathy.

*Resolved*, That a copy of these resolutions be published in the Victoria papers and in THE TEXAS MEDICAL JOURNAL.

Committee.

W. L. WARD, M. D.

W. A. RAPE, M. D.

E. M. SHAW, M. D.

D. H. BRAMAN, M. D.

J. L. LINCECUM, M. D.

H. W. CROUSE, M. D.

F. B. SHIELDS, M. D.

G. L. ROBERTSON, M. D.

THOS. G. DUNCAN, M. D.

---

**Texas Petroleum.**—The JOURNAL is pleased to acknowledge the receipt at the hands of President Prather of a copy of Bulletin No. 5, of the University of Texas, the same being The University of Texas Mineral Survey Bulletin No. 1, on the subject of "Texas Petroleum." I have read it with great interest. It is expected material will be collected for a similar bulletin upon the gold, silver, copper, lead and zinc prospects and mines west of the Pecos river by about the first of next year. Following this will probably be issued a bulletin upon the extent and utilization of Texas deposits of cement rock, sulphur, asphalt rock, clay products, building stones, etc. The University of Texas Mineral Survey is now prepared to undertake all kinds of chemical analyses, assays, etc., and all communications and inquiries should be addressed to "Dr. Wm. B. Phillips, The University of Texas Mineral Survey, Austin, Texas."

